# Supplementary figures and images for: The Listeria monocytogenes persistence factor ClpL is a potent stand-alone disaggregase
Source: eLife. 2024 Apr 10;12:RP92746. doi: 10.7554/eLife.92746 (PMC11006417; doi:10.7554/eLife.92746)

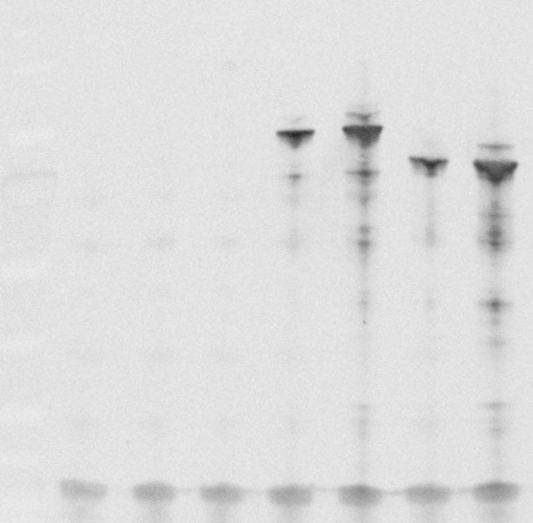

Supplement: Figure 3—figure supplement 2—source data 1. [file elife-92746-fig3-figsupp2-data1.zip › Figure 3-figure supplement 2-source data 1/Figure 3-figure supplement 2C_WB_source data 1.tiff]

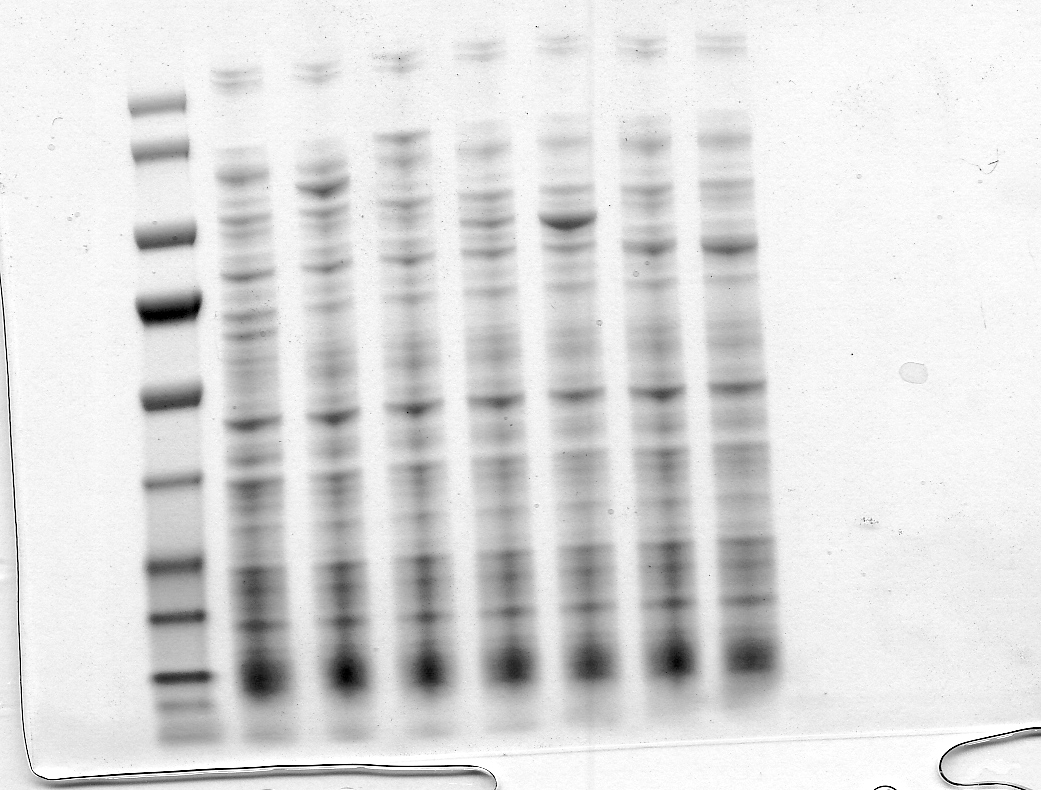

Supplement: Figure 3—figure supplement 2—source data 1. [file elife-92746-fig3-figsupp2-data1.zip › Figure 3-figure supplement 2-source data 1/Figure 3-figure supplement 2B_SDSgel_source data 1.tif]

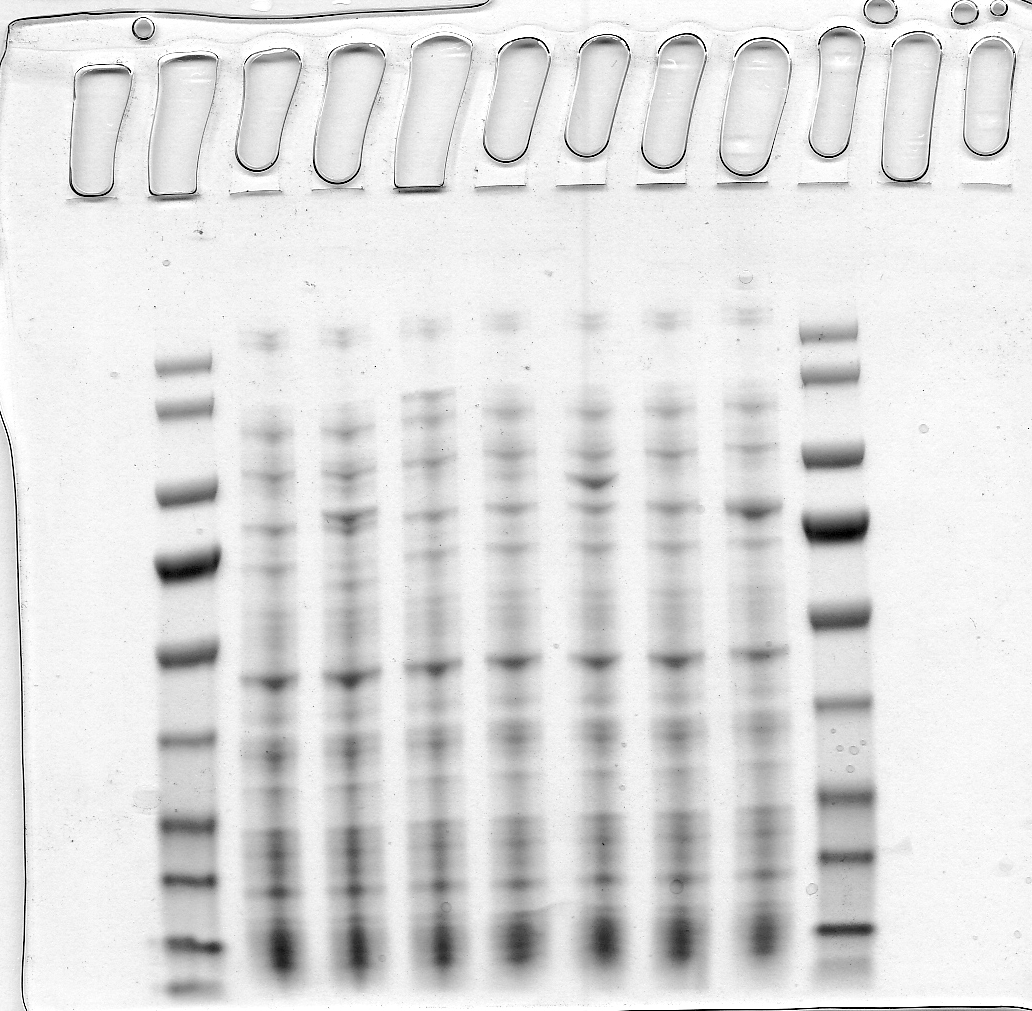

Supplement: Figure 3—figure supplement 2—source data 1. [file elife-92746-fig3-figsupp2-data1.zip › Figure 3-figure supplement 2-source data 1/Figure 3-figure supplement 2C_SDSgel_source data 1.tif]

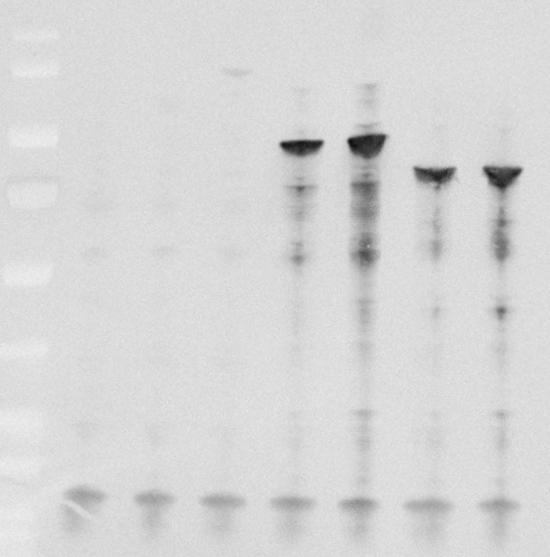

Supplement: Figure 3—figure supplement 2—source data 1. [file elife-92746-fig3-figsupp2-data1.zip › Figure 3-figure supplement 2-source data 1/Figure 3-figure supplement 2B_WB_source data 1.tiff]

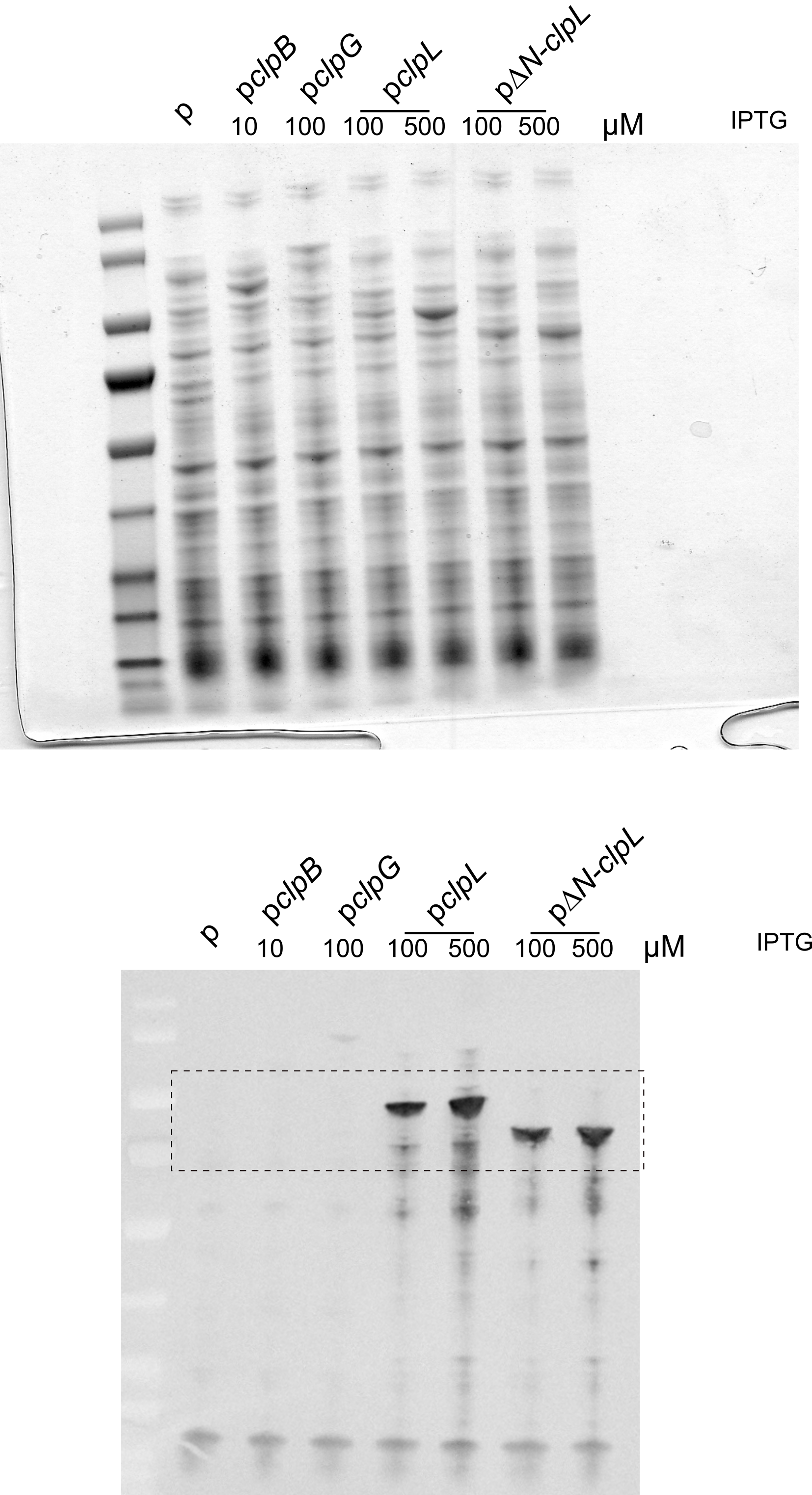

Supplement: Figure 3—figure supplement 2—source data 2. [file elife-92746-fig3-figsupp2-data2.zip › Figure 3-figure supplement 2-source data 2/Figure 3-figure supplement 2B_source data 2.png]

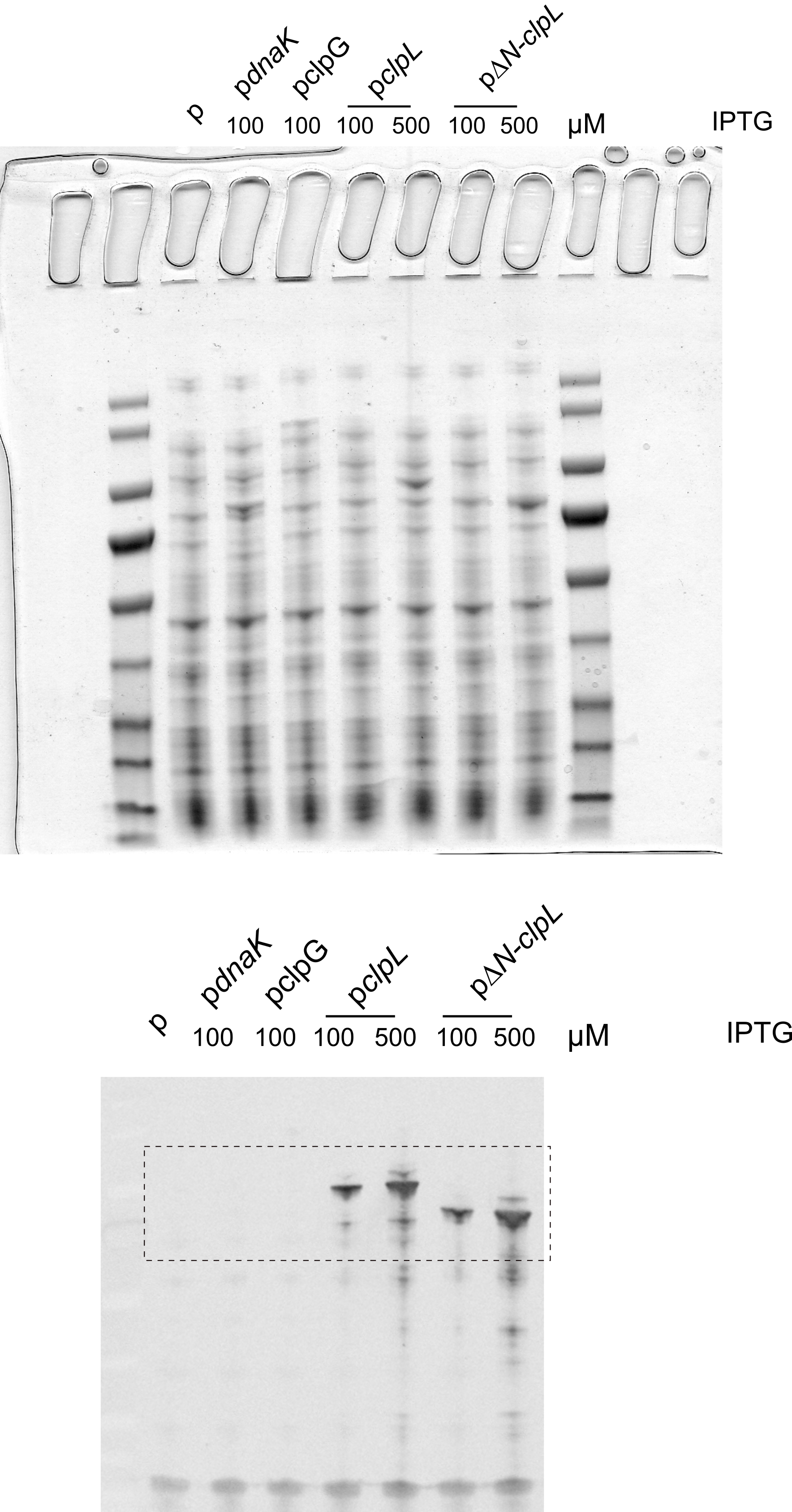

Supplement: Figure 3—figure supplement 2—source data 2. [file elife-92746-fig3-figsupp2-data2.zip › Figure 3-figure supplement 2-source data 2/Figure 3-figure supplement 2C_source data 2.png]

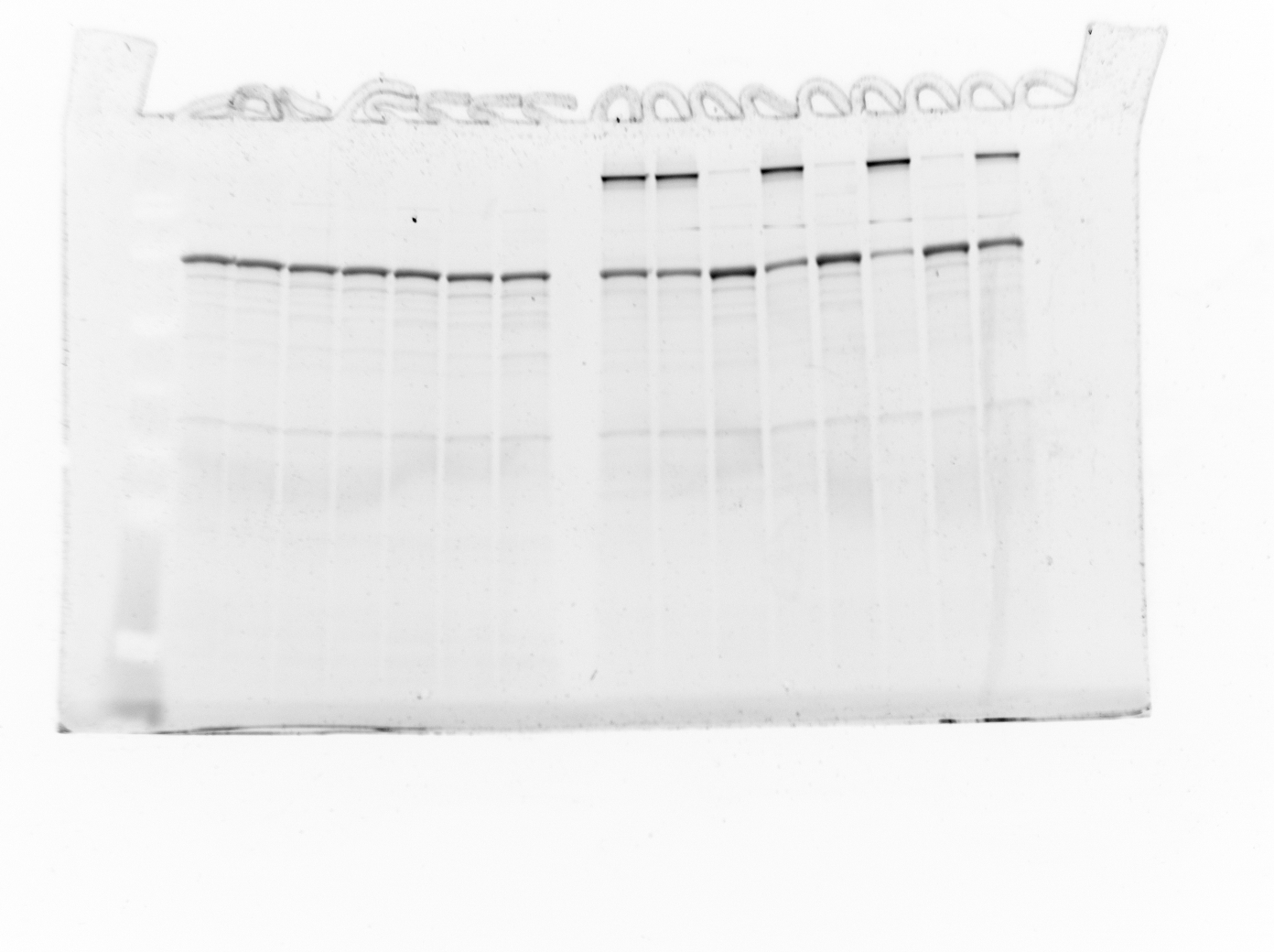

Supplement: Figure 6—source data 1. [file elife-92746-fig6-data1.zip › Figure 6-source data 1/Figure 6F - source data 1.tif]

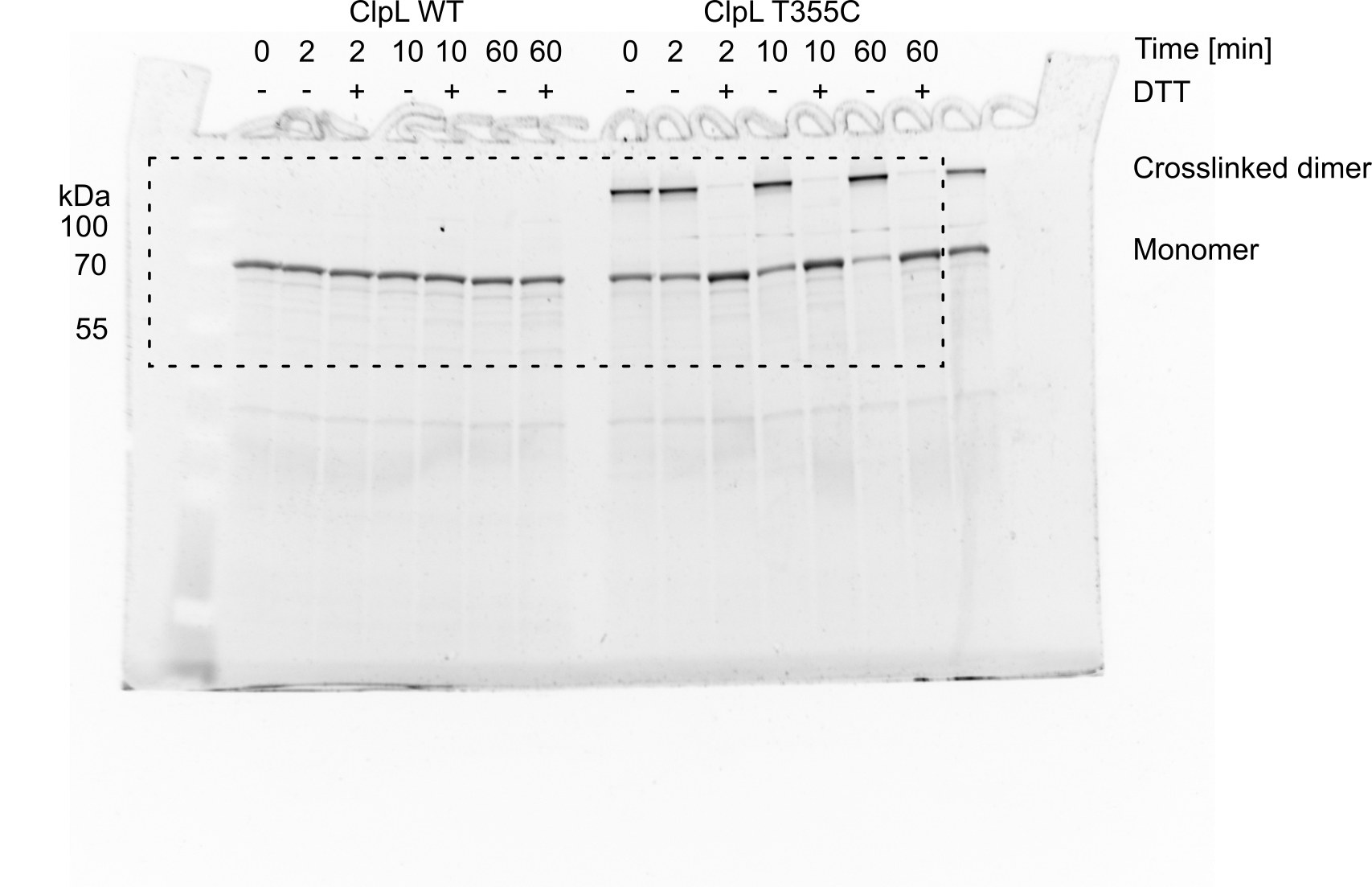

Supplement: Figure 6—source data 2. [file elife-92746-fig6-data2.zip › Figure 6-source data 2/Figure 6F - source data 2.tiff]

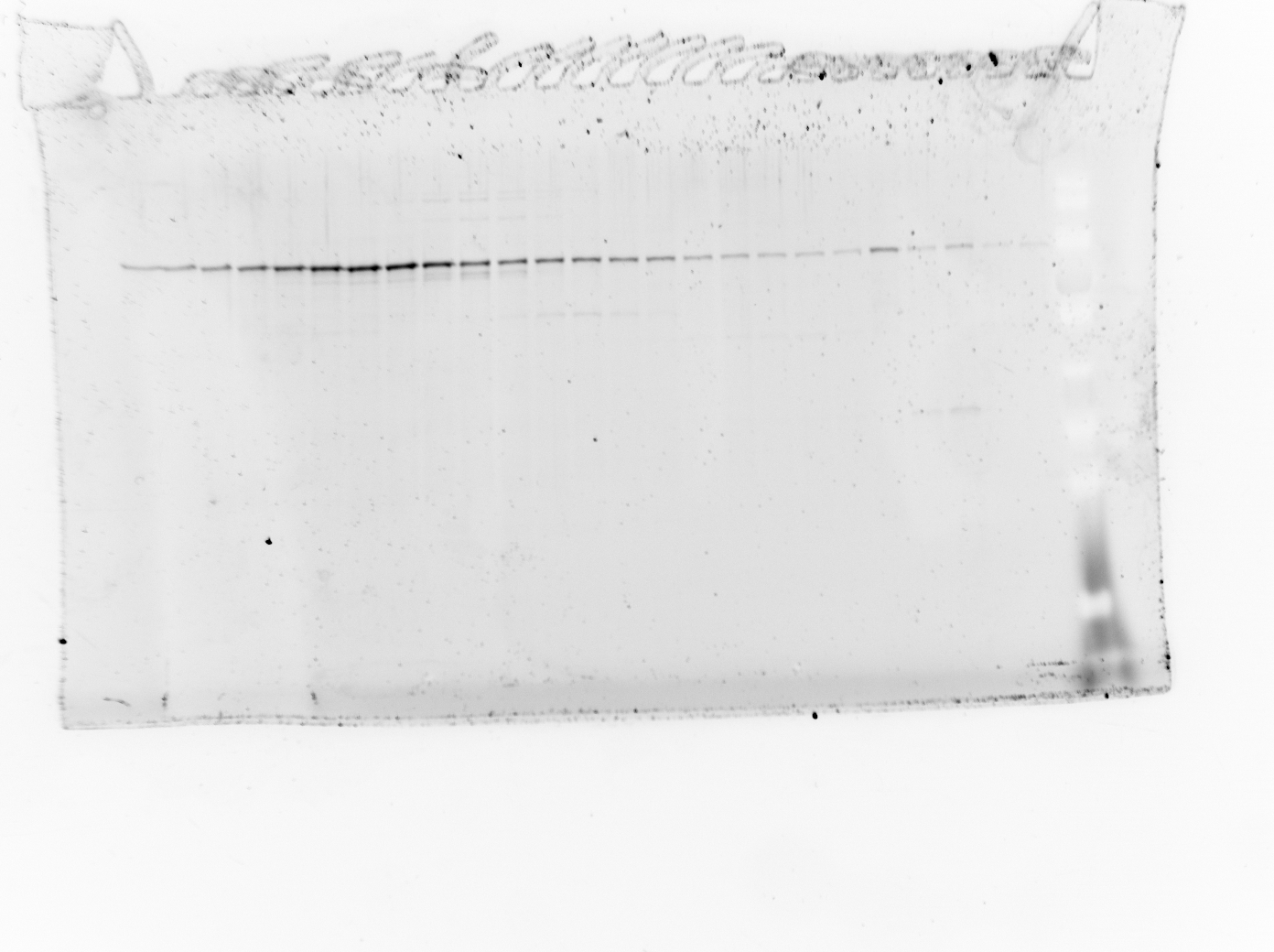

Supplement: Figure 6—figure supplement 1—source data 1. [file elife-92746-fig6-figsupp1-data1.zip › Figure 6-figure supplement 1-source data 1/Figure 6-figure supplement 1A - source data 1j ClpL E352A.tif]

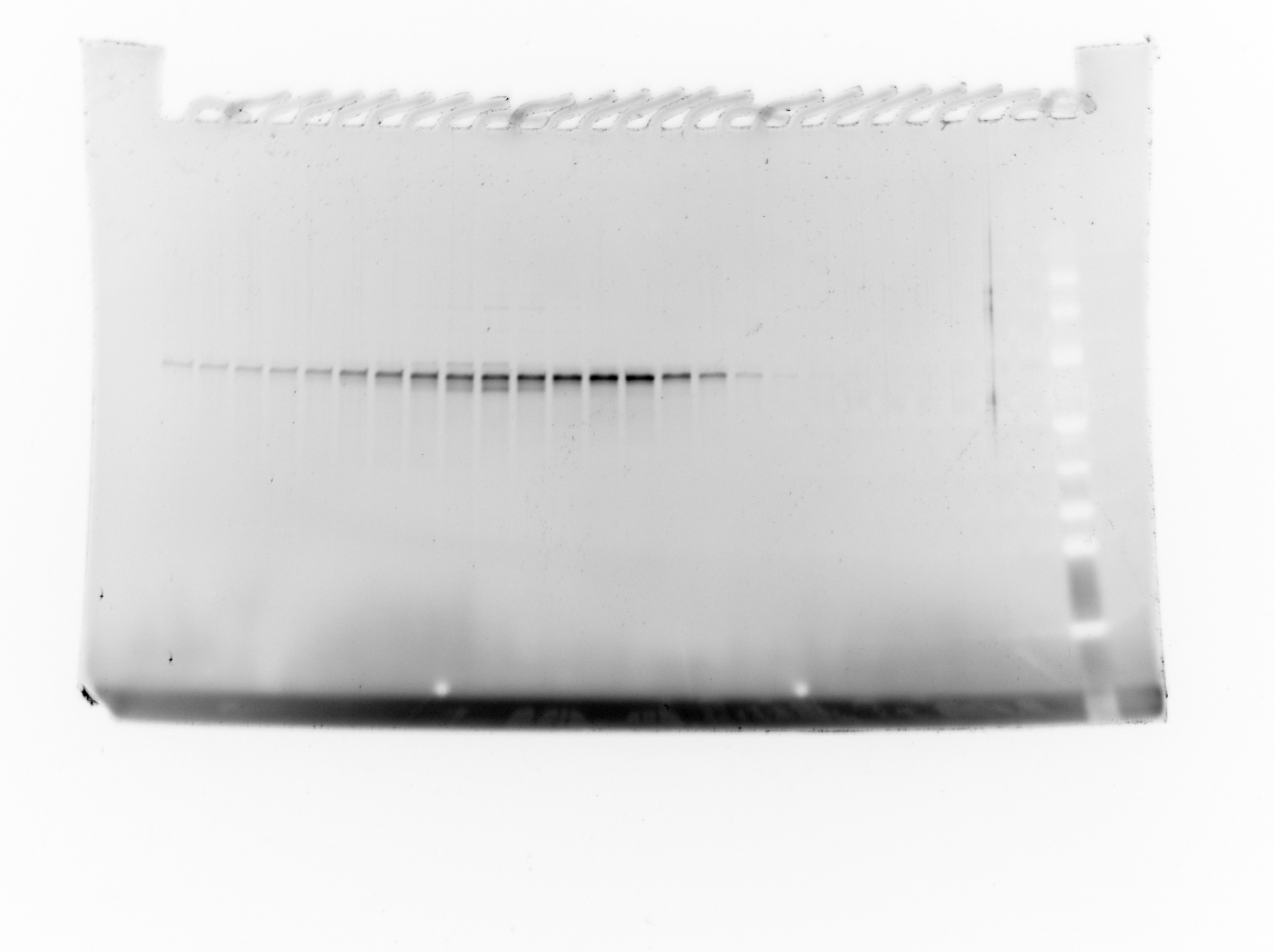

Supplement: Figure 6—figure supplement 1—source data 1. [file elife-92746-fig6-figsupp1-data1.zip › Figure 6-figure supplement 1-source data 1/Figure 6-figure supplement 1A - source data 1e ClpL AB.tif]

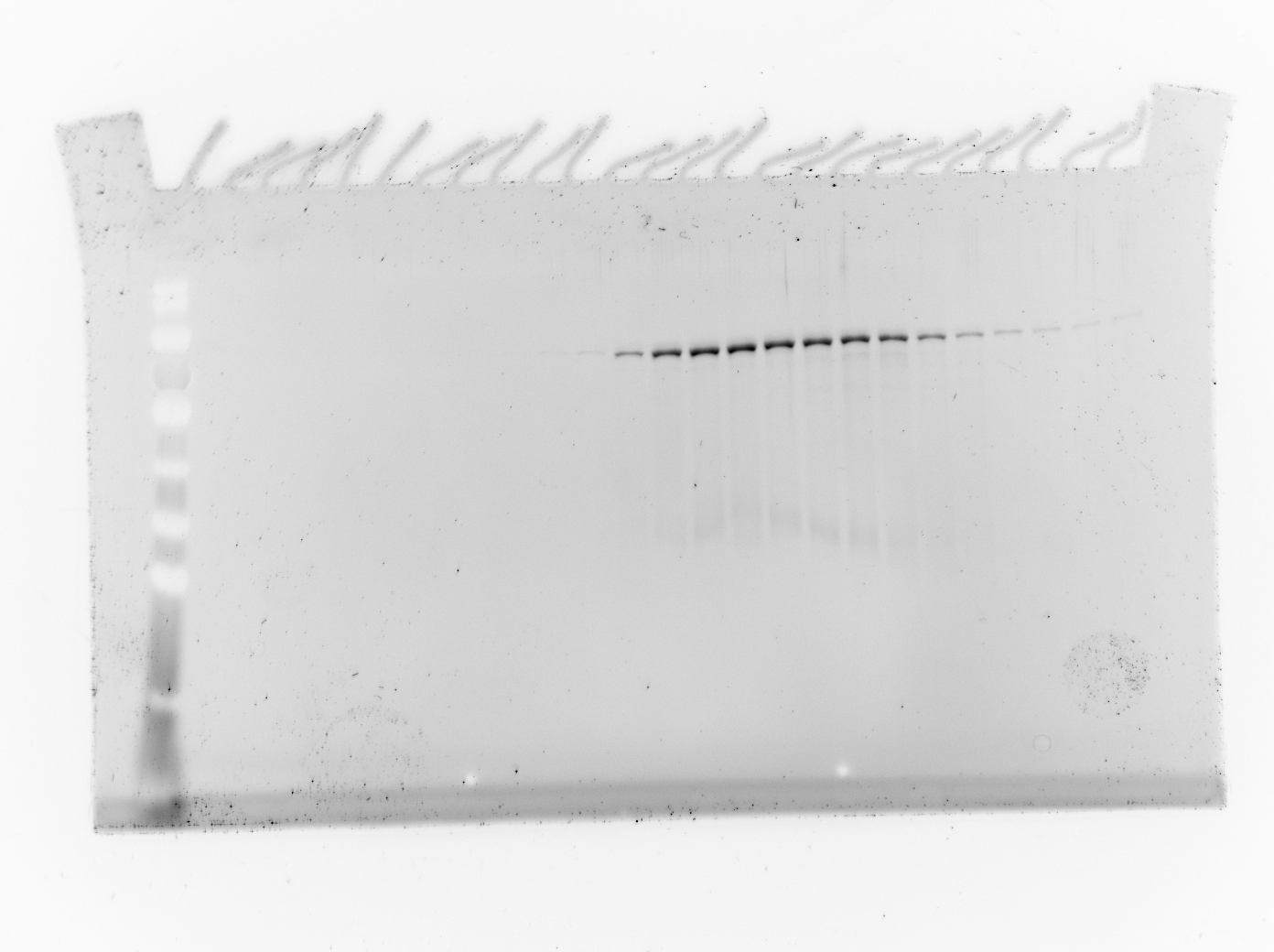

Supplement: Figure 6—figure supplement 1—source data 1. [file elife-92746-fig6-figsupp1-data1.zip › Figure 6-figure supplement 1-source data 1/Figure 6-figure supplement 1A - source data 1f ClpL AC.tif]

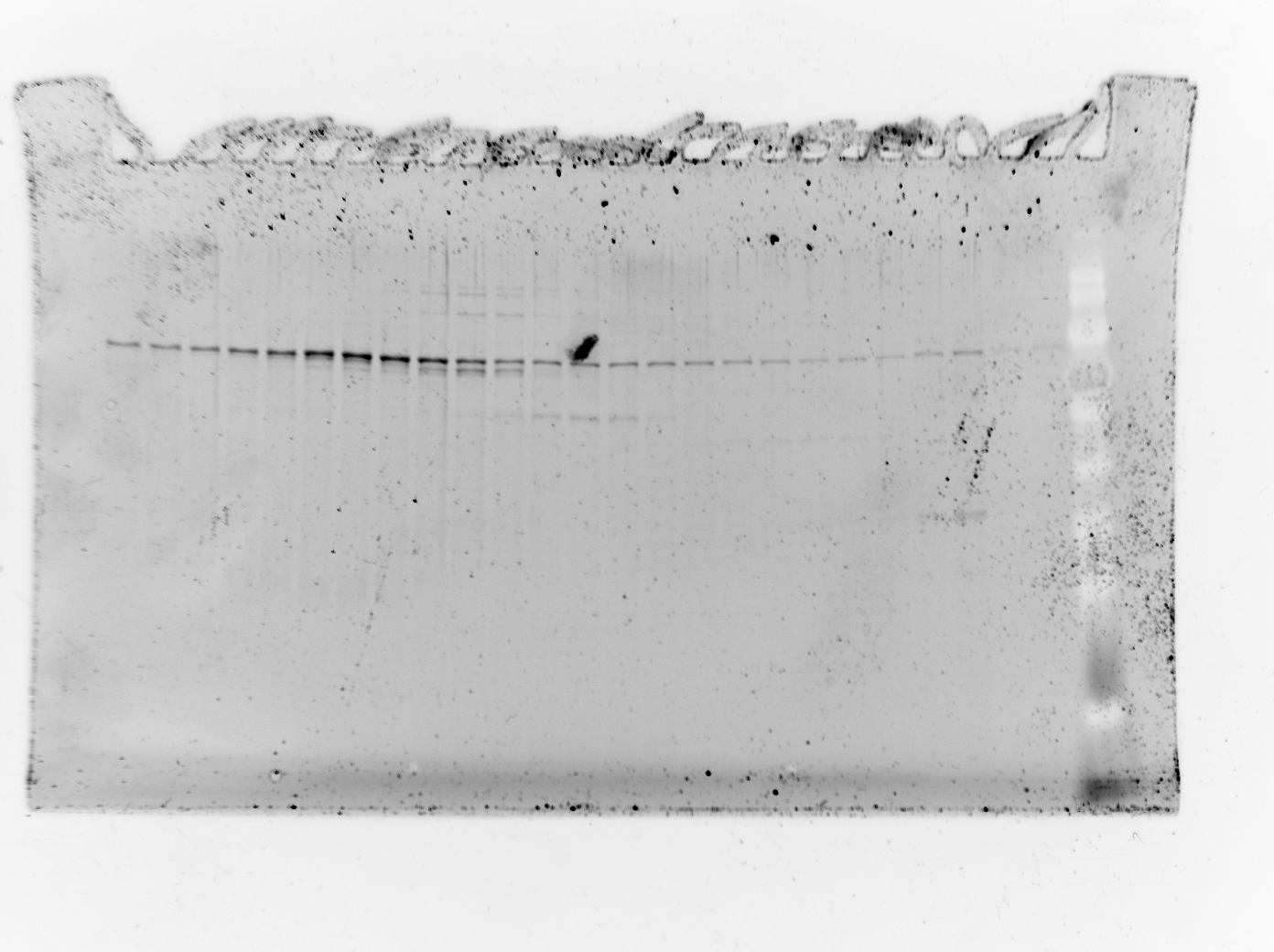

Supplement: Figure 6—figure supplement 1—source data 1. [file elife-92746-fig6-figsupp1-data1.zip › Figure 6-figure supplement 1-source data 1/Figure 6-figure supplement 1A - source data 1i ClpL F354A.tif]

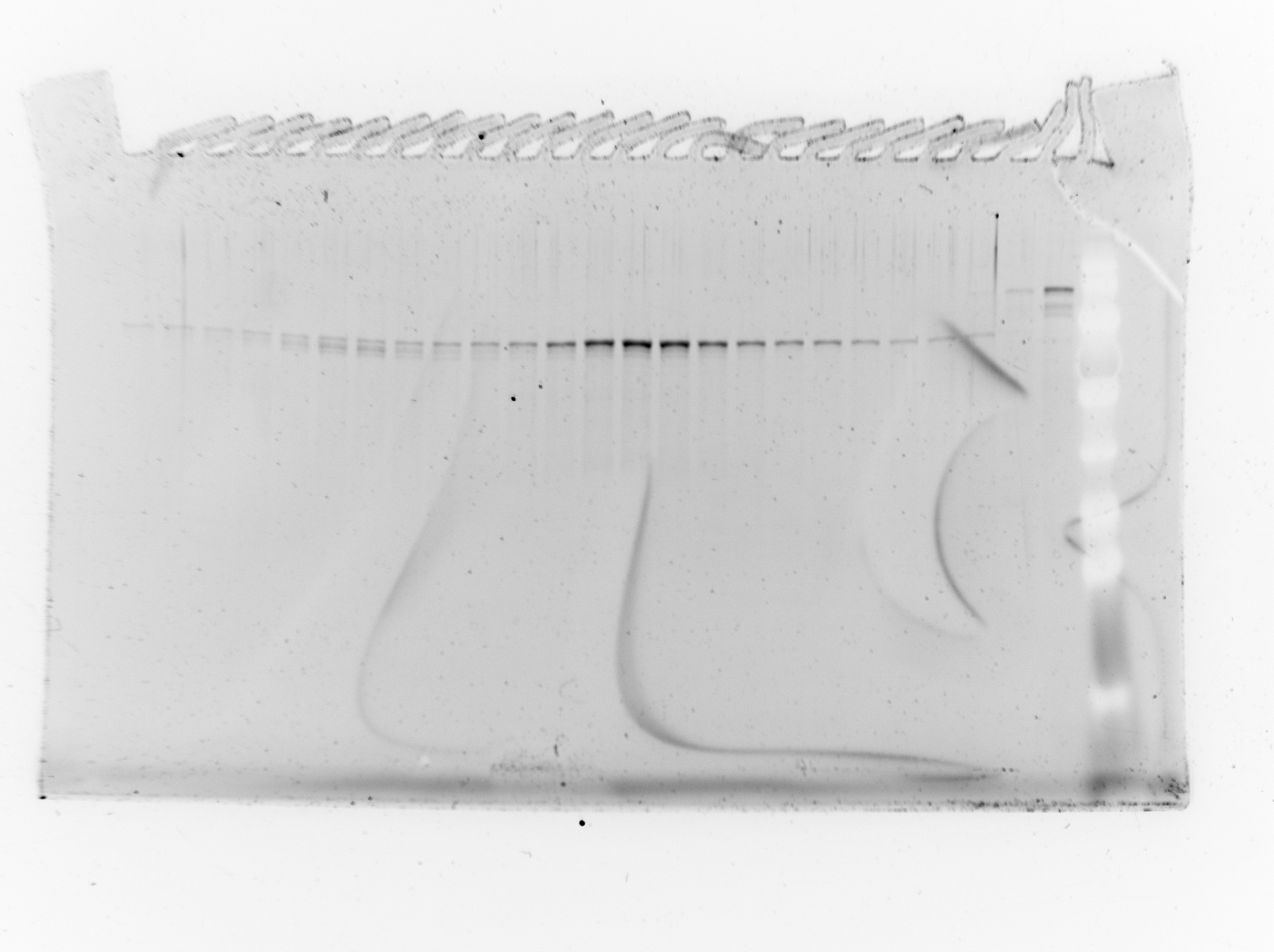

Supplement: Figure 6—figure supplement 1—source data 1. [file elife-92746-fig6-figsupp1-data1.zip › Figure 6-figure supplement 1-source data 1/Figure 6-figure supplement 1A - source data 1a ClpL WT.tif]

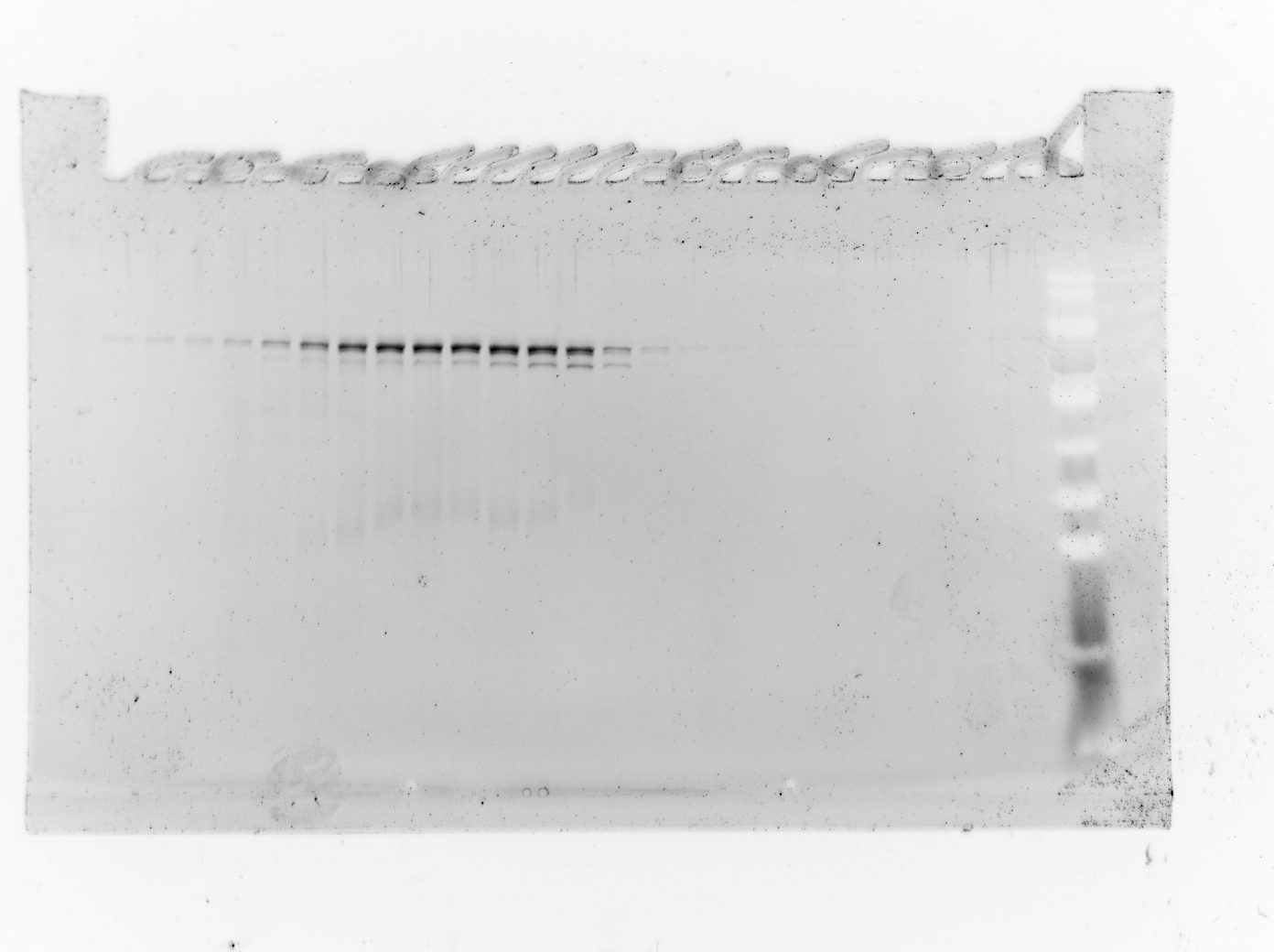

Supplement: Figure 6—figure supplement 1—source data 1. [file elife-92746-fig6-figsupp1-data1.zip › Figure 6-figure supplement 1-source data 1/Figure 6-figure supplement 1A - source data 1h ClpL Aro-Ala.tif]

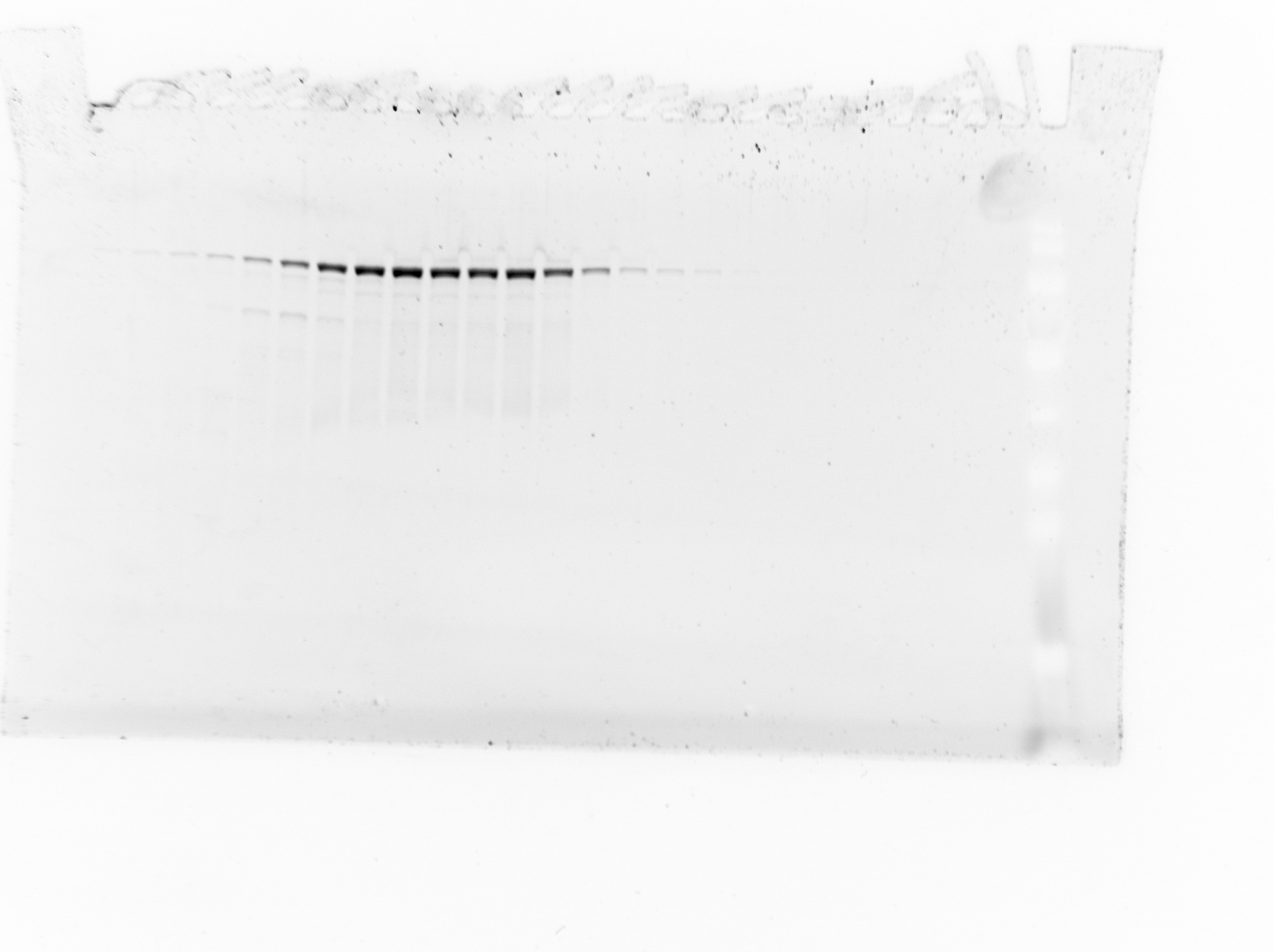

Supplement: Figure 6—figure supplement 1—source data 1. [file elife-92746-fig6-figsupp1-data1.zip › Figure 6-figure supplement 1-source data 1/Figure 6-figure supplement 1A - source data 1b ClpB DWB.tif]

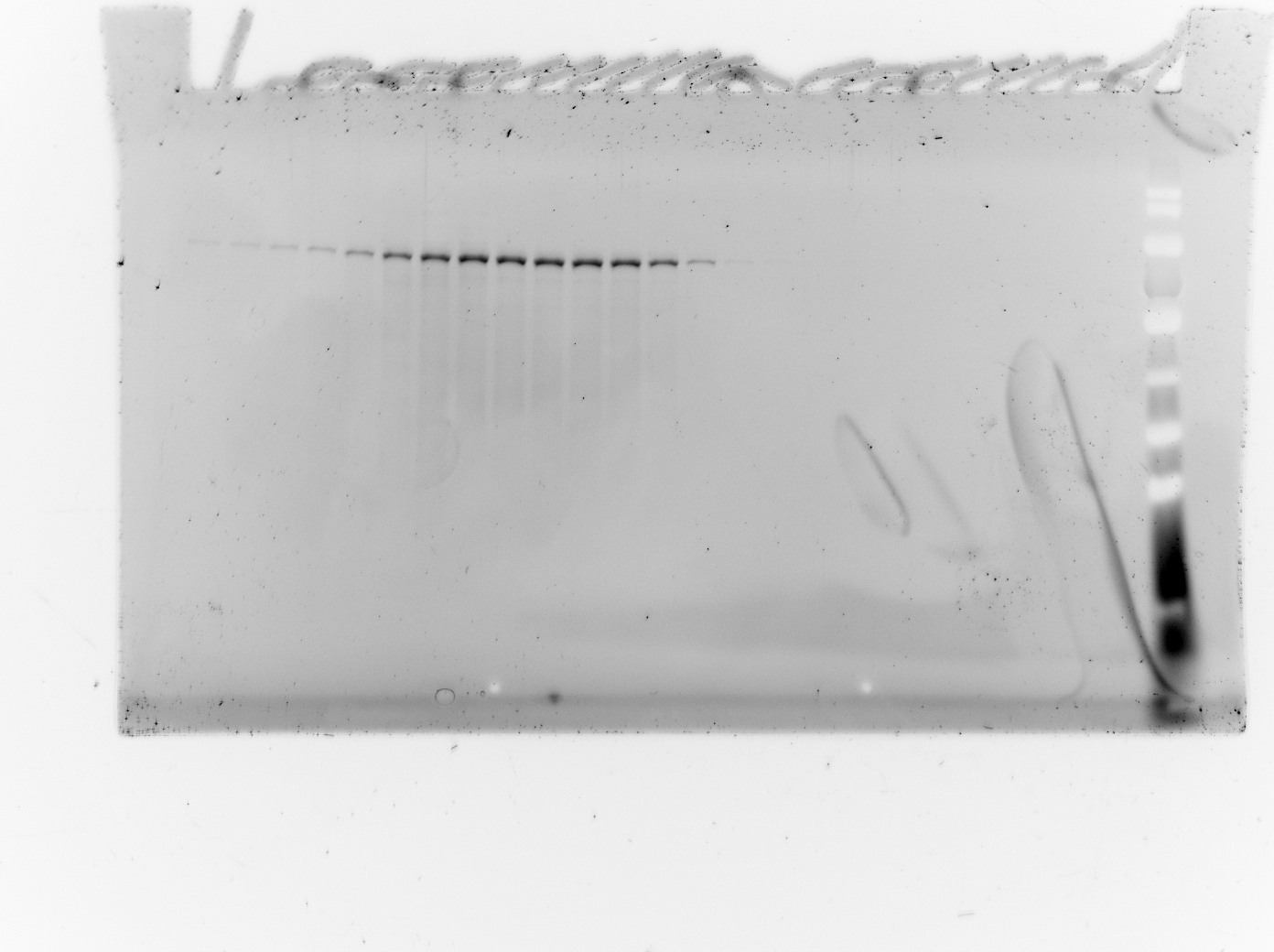

Supplement: Figure 6—figure supplement 1—source data 1. [file elife-92746-fig6-figsupp1-data1.zip › Figure 6-figure supplement 1-source data 1/Figure 6-figure supplement 1A - source data 1g ClpL AC.tif]

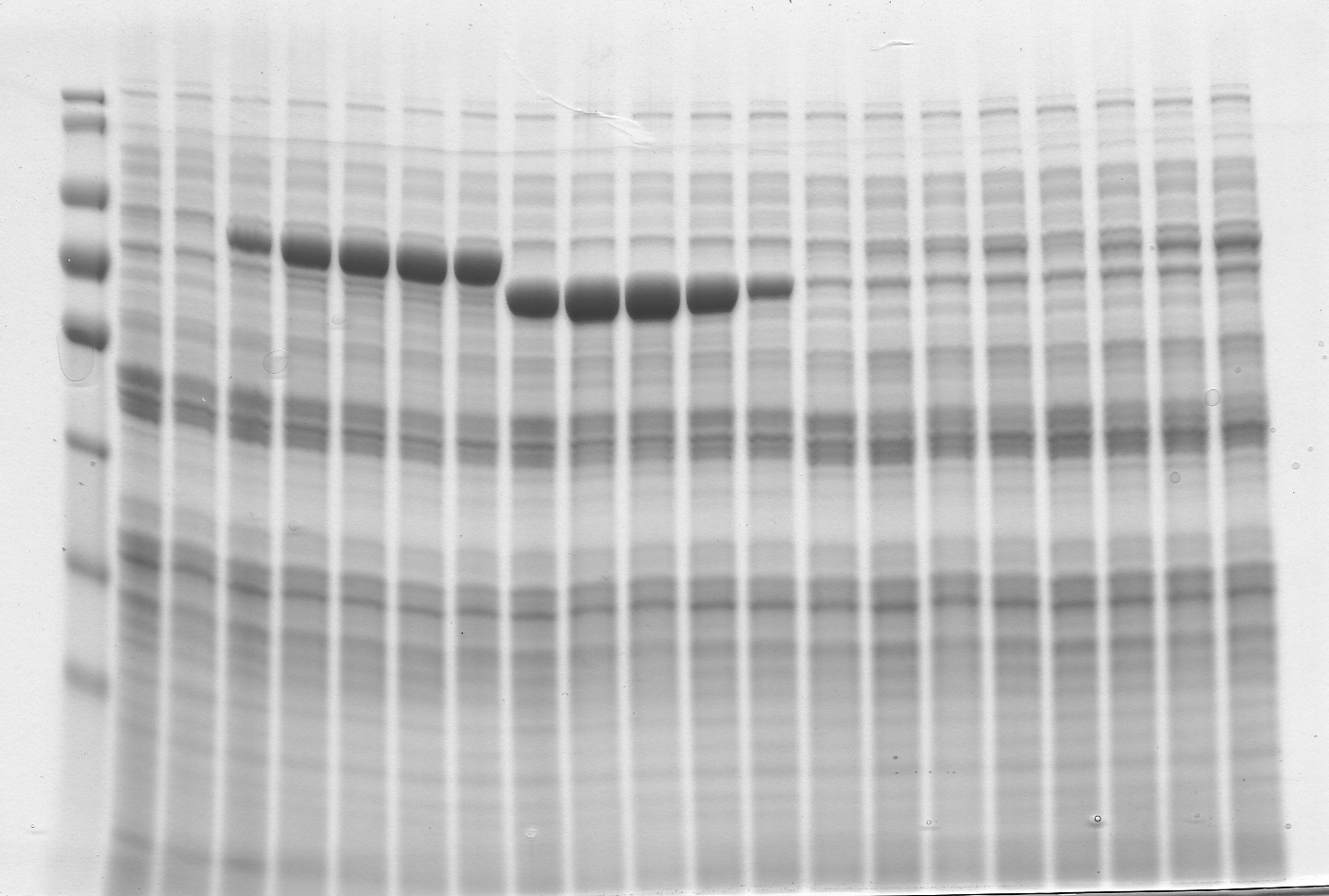

Supplement: Figure 6—figure supplement 3—source data 1. [file elife-92746-fig6-figsupp3-data1.zip › Figure 6-figure supplement 3-source data 1/Figure 6-figure supplement 3A_source data 1.tiff]

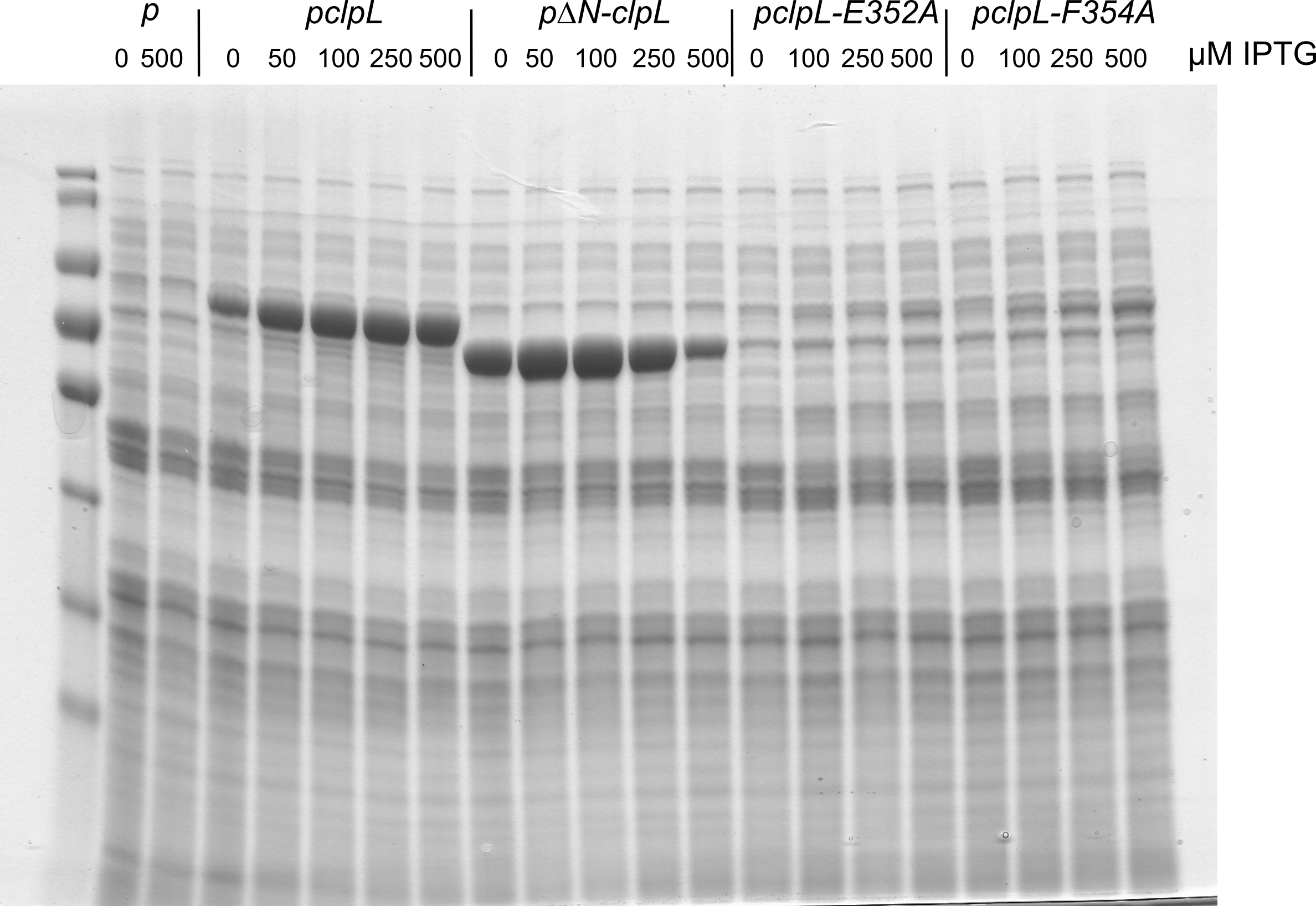

Supplement: Figure 6—figure supplement 3—source data 2. [file elife-92746-fig6-figsupp3-data2.zip › Figure 6-figure supplement 3-source data 2/Figure 6-figure supplement 3A_source data 2.png]
