# Supplementary material for: The Listeria monocytogenes persistence factor ClpL is a potent stand-alone disaggregase
Source: eLife. 2024 Apr 10;12:RP92746. doi: 10.7554/eLife.92746 (PMC11006417; doi:10.7554/eLife.92746)
Supplement: Supplementary file 1. [file elife-92746-supp1.docx]

**Supplementary File 1: strains and plasmids used in this study**

| **Strain** | **Description** | **Source or reference** |
| --- | --- | --- |
| *E. coli* XL1 blue | *recA1 endA1 gyrA96 thi-1 hsdR1 supE44 relA1 lac* [F’ *proAB* *lacI^q^ ΔM15* Tn10 (Tcr)] | Stratagene |
| *E. coli* BL21 | *F- ompT lon hsdSB gal dcm λ* (DE3) | Novagen |
| *E. coli* *ΔclpB* | MC1000 Δ*clpB*::Km | (1) |
| *E. coli dnak103* | MC4100 *dnak103* | (1) |
| **Plasmid** | **Description** | **Source or reference** |
| pET24a-*clpL* | Vector for IPTG-inducible expression of *clpL* in *E.coli* BL21 cells, fused C-terminal 6xHis-tag | This study |
| pET24a*-clpL-∆N* | Vector for IPTG-inducible expression of *clpL-∆N* in *E.coli* BL21 cells, fused C-terminal 6xHis-tag | This study |
| pET24a*-Lm_clpB* | Vector for IPTG-inducible expression of *Lm clpB* in *E.coli* BL21 cells, fused C-terminal 6xHis-tag | This study |
| pET24a*-Lm_DnaK* | Vector for IPTG-inducible expression of *Lm_DnaK* in *E.coli* BL21 cells, fused C-terminal 6xHis-tag | This study |
| pET24a*-Lm_DnaJ* | Vector for IPTG-inducible expression of *Lm_DnaJ* in *E.coli* BL21 cells, fused C-terminal 6xHis-tag | This study |
| pET24a*-Lm_GrpE* | Vector for IPTG-inducible expression of *Lm_GrpE* in *E.coli* BL21 cells, fused C-terminal 6xHis-tag | This study |
| pET24a*-clpL-Patch_A* | Vector for IPTG-inducible expression of *clpL-Patch_A* in *E.coli* BL21 cells, fused C-terminal 6xHis-tag | This study |
| pET24a*-clpL-Patch_B* | Vector for IPTG-inducible expression of *clpL-Patch_B* in *E.coli* BL21 cells, fused C-terminal 6xHis-tag | This study |
| pET24a*-clpL-Patch_C* | Vector for IPTG-inducible expression of *clpL-Patch_C* in *E.coli* BL21 cells, fused C-terminal 6xHis-tag | This study |
| pET24a*-clpL-Patch_D* | Vector for IPTG-inducible expression of *clpL-Patch_D* in *E.coli* BL21 cells, fused C-terminal 6xHis-tag | This study |
| pET24a*-clpL-Patch_E* | Vector for IPTG-inducible expression of *clpL-Patch_E* in *E.coli* BL21 cells, fused C-terminal 6xHis-tag | This study |
| pET24a*-clpL-Patch_AB* | Vector for IPTG-inducible expression of *clpL-Patch_AB* in *E.coli* BL21 cells, fused C-terminal 6xHis-tag | This study |
| pET24a*-clpL-Patch_AC* | Vector for IPTG-inducible expression of *clpL-Patch_AC* in *E.coli* BL21 cells, fused C-terminal 6xHis-tag | This study |
| pET24a*-clpL-Patch_BC* | Vector for IPTG-inducible expression of *clpL-Patch_BC* in *E.coli* BL21 cells, fused C-terminal 6xHis-tag | This study |
| pET24a*-clpL_all_NQ🡪Ala* | Vector for IPTG-inducible expression of *clpL-Patch_all_NQ🡪Ala*  in *E.coli* BL21 cells, fused C-terminal 6xHis-tag | This study |
| pET24a*-clpL-Patch_all_Aro🡪Ala* | Vector for IPTG-inducible expression of *clpL-Patch_all_Aro🡪Ala* in *E.coli* BL21 cells, fused C-terminal 6xHis-tag | This study |
| pET24a*-clpL-Y36A* | Vector for IPTG-inducible expression of *clpL-Y36A* in *E.coli* BL21 cells, fused C-terminal 6xHis-tag | This study |
| pET24a*-clpL-F48A* | Vector for IPTG-inducible expression of *clpL-F48A* in *E.coli* BL21 cells, fused C-terminal 6xHis-tag | This study |
| pET24a*-clpL-Y51A* | Vector for IPTG-inducible expression of *clpL-Y51A* in *E.coli* BL21 cells, fused C-terminal 6xHis-tag | This study |
| pET24a*-clpL-NTD61* | Vector for IPTG-inducible expression of *clpL_NTD61* in *E.coli* BL21 cells, fused C-terminal 6xHis-tag | This study |
| pET24a*-clpL-NTD78* | Vector for IPTG-inducible expression of *clpL_NTD78* in *E.coli* BL21 cells, fused C-terminal 6xHis-tag | This study |
| pCA528-*clpL-E352A* | Vector for IPTG-inducible expression of *clpL-E352A* in *E.coli* BL21 cells, fused cleavable N-terminal 6xHis-SUMO-tag | This study |
| pCA528-*clpL-F354A* | Vector for IPTG-inducible expression of *clpL-F354A* in *E.coli* BL21 cells, fused cleavable N-terminal 6xHis-SUMO-tag | This study |
| pCA528-*clpL-T355C* | Vector for IPTG-inducible expression of *clpL-T355C* in *E.coli* BL21 cells, fused cleavable N-terminal 6xHis-SUMO-tag | This study |
| pC6Amp∆BsaI-*L_N_-ClpB** | Vector for IPTG-inducible expression of *L_N_-ClpB** in *E.coli* BL21 cells, fused cleavable N-terminal 6xHis-SUMO-tag | This study |
| pC6Amp∆BsaI-*L_N_-ClpB*-E199A-E598A* | Vector for IPTG-inducible expression of *L_N_-ClpB*- E199A-E598A* in *E.coli* BL21 cells, fused cleavable N-terminal 6xHis-SUMO-tag | This study |
| pC6Amp∆BsaI-*L_N_-ClpB*-Patch_A* | Vector for IPTG-inducible expression of *L_N_-ClpB*-Patch_A* in *E.coli* BL21 cells, fused cleavable N-terminal 6xHis-SUMO-tag | This study |
| pC6Amp∆BsaI-*L_N_-ClpB*-Patch_AB* | Vector for IPTG-inducible expression of *L_N_-ClpB*-Patch_AB* in *E.coli* BL21 cells, fused cleavable N-terminal 6xHis-SUMO-tag | This study |
| pC6Amp∆BsaI-*L_N_-ClpB*-Patch_C* | Vector for IPTG-inducible expression of *L_N_-ClpB*-Patch_C* in *E.coli* BL21 cells, fused cleavable N-terminal 6xHis-SUMO-tag | This study |
| pC6Amp∆BsaI-*L_N_-ClpB*-Y36A* | Vector for IPTG-inducible expression of *L_N_-ClpB*-Y36A* in *E.coli* BL21 cells, fused cleavable N-terminal 6xHis-SUMO-tag | This study |
| pC6Amp∆BsaI-*L_N_-ClpB*-F48A* | Vector for IPTG-inducible expression of *L_N_-ClpB*-F48A* in *E.coli* BL21 cells, fused cleavable N-terminal 6xHis-SUMO-tag | This study |
| pC6Amp∆BsaI-*L_N_-ClpB*-Y51A* | Vector for IPTG-inducible expression of *L_N_-ClpB*-Y51A* in *E.coli* BL21 cells, fused cleavable N-terminal 6xHis-SUMO-tag | This study |
| pUHE21-2fd12 | Empty Vector control for *in vivo* assays | This study |
| pUHE21-*clpG_GI_* | Vector for IPTG-inducible expression of *clpG_GI_* in *E.coli* *∆clpB* cells | adjust |
| pUHE21-*Ec* *clpB* | Vector for IPTG-inducible expression of *Ec* *clpB* in *E.coli* *∆clpB* cells | adjust |
| pDS56-*clpL* | Vector for IPTG-inducible expression of *clpL* in *E.coli* *ΔclpB* cells and *E. coli dnak103* cells | This study |
| pUHE21-*clpL-∆N* | Vector for IPTG-inducible expression of *clpL* in *E.coli* *ΔclpB* cells and *E. coli dnak103* cells | This study |
| pDS56-*clpL-E352A* | Vector for IPTG-inducible expression of *clpL* in *E.coli* *ΔclpB* cells | This study |
| pDS56-*clpL-F354A* | Vector for IPTG-inducible expression of *clpL* in *E.coli* *ΔclpB* cells | This study |
| pUHE21-*Ec* dnaK | Vector for IPTG-inducible expression of *Ec dnaK* in *E.coli* *dnak103* cells | (1) |
| pET24a-*clpG_GI_* | Vector for IPTG-inducible expression of *clpG_GI_* in *E.coli* BL21 cells, fused C-terminal 6xHis-tag | adjust |
| pDS56-*Ec* *clpB* | Vector for IPTG-inducible expression of *Ec* *clpB* in *E.coli* *∆clpB* cells, fused C-terminal 6xHis-tag | (1) |
| pUHE21-*Ec dnaK*-His | Vector for IPTG-inducible expression of *Ec dnaK* in *E.coli* *∆clpB* cells, fused C-terminal 6xHis-tag | (1) |
| pCA528-*Ec dnaJ* | Vector for IPTG-inducible expression of *Ec dnaJ* in *E.coli* BL21 cells, fused cleavable N-terminal 6xHis-SUMO-tag | (1) |
| pCA528-*Ec grpE* | Vector for IPTG-inducible expression of *Ec grpE* in *E.coli* BL21 cells, fused cleavable N-terminal 6xHis-SUMO-tag | (1) |
| pDS56-*Ec* ∆N-ClpB-K476C | Vector for IPTG-inducible expression of *Ec* *∆N-clpB-K476C*  in *E.coli* *∆clpB* cells, fused C-terminal 6xHis-tag | (2) |
| pDS56-*luciferase* | Vector for IPTG-inducible expression of *luciferase* in *E.coli* *∆clpB* cells, fused N-terminal 6xHis-tag | (3) |
| pDS56-*luciferase-yfp* | Vector for IPTG-inducible expression of *luciferase-YFP* in *E.coli* *∆clpB* cells, fused N-terminal 6xHis-tag | (3) |

**References**

1. Lee, C., Franke, K. B., Kamal, S. M., Kim, H., Lunsdorf, H., Jager, J., Nimtz, M., Trcek, J., Jansch, L., Bukau, B., Mogk, A., and Romling, U. (2018) Stand-alone ClpG disaggregase confers superior heat tolerance to bacteria. *Proc Natl Acad Sci U S A* **115**, E273-E282

2. Oguchi, Y., Kummer, E., Seyffer, F., Berynskyy, M., Anstett, B., Zahn, R., Wade, R. C., Mogk, A., and Bukau, B. (2012) A tightly regulated molecular toggle controls AAA+ disaggregase. *Nat Struct Mol Biol* **19**, 1338-1346

3. Haslberger, T., Zdanowicz, A., Brand, I., Kirstein, J., Turgay, K., Mogk, A., and Bukau, B. (2008) Protein disaggregation by the AAA+ chaperone ClpB involves partial threading of looped polypeptide segments. *Nat Struct Mol Biol* **15**, 641-650
