# Supplementary material for: The Listeria monocytogenes persistence factor ClpL is a potent stand-alone disaggregase
Source: eLife. 2024 Apr 10;12:RP92746. doi: 10.7554/eLife.92746 (PMC11006417; doi:10.7554/eLife.92746)
Supplement: Supplementary file 2. [file elife-92746-supp2.docx]

**Supplementary File 2: number of replicates**

Number of replicates by figure and data set, listed in plot order (left-to-right or top-down, according to labels)

| **Figure** | **Data 1** | **Data 2** | **Data 3** | **Data 4** | **Data 5** | **Data 6** | **Data 7** | **Data 8** | **Data 9** |
| --- | --- | --- | --- | --- | --- | --- | --- | --- | --- |
| Fig. 1B1 | 58 |  |  |  |  |  |  |  |  |
| Fig. 1B2 | 18 | 18 | 8 | 4 | 10 | 4 | 4 |  |  |
| Fig. 1C | 3 | 4 | 3 | 3 | 3 |  |  |  |  |
| Fig. 1E | 6 | 5 |  |  |  |  |  |  |  |
| Fig. 2B | 3 | 3 | 4 | 4 | 3 |  |  |  |  |
| Fig. 2C | 4 | 4 | 4 | 4 |  |  |  |  |  |
| Fig. 2D | 3 | 3 | 3 |  |  |  |  |  |  |
| Fig. 3A | 8 | 8 | 5 | 5 |  |  |  |  |  |
| Fig. 3B | 3 | 3 |  |  |  |  |  |  |  |
| Fig. 3C | 4 | 4 | 4 | 4 | 4 | 4 | 4 |  |  |
| Fig. 3E | 3 | 3 | 3 | 3 | 3 | 3 | 3 |  |  |
| Fig. 3F | 4 | 4 | 4 | 4 | 4 | 4 | 4 |  |  |
| Fig. 4E | 29 | 28 | 4 | 6 | 13 | 3 | 3 | 5 | 20 |
| Fig. 4F | 9 | 9 | 4 | 4 | 7 | 3 | 3 | 3 | 7 |
| Fig. 4G | 3 | 3 | 3 | 3 |  |  |  |  |  |
| Fig. 5B | 13 | 13 | 13 | 13 | 13 | 13 |  |  |  |
| Fig. 5C | 3 | 3 | 3 | 3 |  |  |  |  |  |
| Fig. 5D | 3 | 3 | 3 | 3 |  |  |  |  |  |
| Fig. 6C* | 5233 | 18314 | 1900 | 14215 | 7074 | 11765 |  |  |  |
| Fig. 6D | 10 | 6 | 4 | 4 |  |  |  |  |  |
| Fig. 6E | 8 | 5 | 3 | 3 |  |  |  |  |  |
| Fig. 6G | 6 | 6 | 6 | 6 | 6 | 6 |  |  |  |
| Fig. 1 – Figure S2A | 3 | 3 | 3 | 3 |  |  |  |  |  |
| Fig. 1 – Figure S2C | 4 | 3 | 4 | 3 |  |  |  |  |  |
| Fig. 1 – Figure S2D | 5 | 5 | 5 | 5 | 5 |  |  |  |  |
| Fig. 1 – Figure S2E (left) | 19 |  |  |  |  |  |  |  |  |
| Fig. 1 – Figure S2E (right) | 3 | 3 | 3 | 3 | 3 | 3 | 3 |  |  |
| Fig. 1 – Figure S2F | 13 | 6 | 4 | 4 | 3 | 3 | 6 | n.d. |  |
| Fig. 1 – Figure S2G | 5 | 5 | 5 |  |  |  |  |  |  |
| Fig. 2 – Figure S1 | 5 | 4 | 4 | 3 | 4 | 4 | 3 |  |  |
| Fig. 3 – Figure S1B | 24 | 19 |  |  |  |  |  |  |  |
| Fig. 3 – Figure S1C | 3 | 3 | 3 | 3 | 3 | 3 |  |  |  |
| Fig. 3 – Figure S1D | 3 | 3 | 3 | 3 | 3 | 3 | 3 |  |  |
| Fig. 3 – Figure S2D | 3 | 3 |  |  |  |  |  |  |  |
| Fig. 4 – Figure S2A (left) | 12 | 9 | 3 | 12 | 3 | 3 | 3 | 7 |  |
| Fig. 4 – Figure S2A (right) | 3 | 3 | 3 | 3 |  |  |  |  |  |
| Fig. 4 – Figure S2B | 24 | 22 | 7 | 7 | 9 | 9 | 9 |  |  |
| Fig. 4 – Figure S2C | 10 | 8 | 5 | 5 | 5 | 5 | 5 |  |  |
| Fig. 4 – Figure S2D (left) | 9 | 9 | 6 | 6 | 3 | 3 | 6 |  |  |
| Fig. 4 – Figure S2D (right) | 7 | 3 | 4 | 4 | 4 |  |  |  |  |
| Fig. 4 – Figure S2E | 14 | 14 | 10 | 10 | 10 | 6 | 6 | 6 |  |
| Fig. 4 – Figure S2F | 3 | 3 | 3 | 3 | 3 | 3 | 3 | 3 |  |
| Fig. 4 – Figure S2G (left) | 3 | 3 | 3 | 3 | 3 |  |  |  |  |
| Fig. 4 – Figure S2G (right) | 7 | 7 | 3 | 3 | 3 | 4 | 4 | 4 |  |
| Fig. 4 – Figure S2H | 3 | 3 | 3 | 3 | 5 | 3 |  |  |  |
| Fig. 5 – Figure S1A | 3 | 3 | 3 | 3 | 3 | 3 |  |  |  |
| Fig. 5 – Figure S1B | 3 | 3 | 3 | 3 |  |  |  |  |  |
| Fig. 5 – Figure S1C (black) | 13 | 13 | 13 | 13 | 13 | 13 | 13 |  |  |
| Fig. 5 – Figure S1C (red) | 13 | 13 | 13 | 13 | 13 | 13 | 13 |  |  |
| Fig. 5 – Figure S1D (red) | 13 | 13 | 13 | 13 | 13 | 13 | 13 |  |  |
| Fig. 5 – Figure S1E (black) | 13 | 13 | 13 | 13 | 13 | 13 | 13 |  |  |
| Fig. 5 – Figure S1E (red) | 13 | 13 | 13 | 13 | 13 | 13 | 13 |  |  |
| Fig. 6 – Figure S1D | 3 | 3 | 3 | 4 | 4 | 4 |  |  |  |
| Fig. 6 – Figure S1E* | 3588 | 7404 | 9655 |  |  |  |  |  |  |
| Fig. 6 – Figure S1F (left) | 6 | 3 | 3 | 3 |  |  |  |  |  |
| Fig. 6 – Figure S1F (right) | 6 | 3 | 3 | 3 |  |  |  |  |  |
| Fig. 6 – Figure S2A (left) | 3 | 3 |  |  |  |  |  |  |  |
| Fig. 6 – Figure S2A (right) | 3 | 3 |  |  |  |  |  |  |  |
| Fig. 6 – Figure S2B | 7 | 7 |  |  |  |  |  |  |  |
| Fig. 6 – Figure S2D | 6 | 6 | 6 | 6 | 6 | 6 |  |  |  |

*(total particles, not replicates)
